# Effects of deep frying and baking on the quality attributes, water distribution, and flavor characteristics of duck jerky

**DOI:** 10.3389/fnut.2024.1309924

**Published:** 2024-02-07

**Authors:** Yamin Pei, Xingyue Guo, Xionghui Shu, Yahong Han, Youwei Ai, Hongxun Wang, Wenfu Hou

**Affiliations:** ^1^College of Food Science and Engineering, Wuhan Polytechnic University, Wuhan, Hubei, China; ^2^Key Laboratory for Deep Processing of Major Grain and Oil, Ministry of Education, Wuhan, Hubei, China; ^3^Hubei Key Laboratory for Processing and Transformation of Agricultural Products, Wuhan, Hubei, China; ^4^Hubei Jingchu Specialty Food Industry Technology Research Institute, Jingzhou, Hubei, China; ^5^College of Biological and Pharmaceutical Engineering, Wuhan Polytechnic University, Wuhan, Hubei, China

**Keywords:** duck jerky, processing methods, deep frying, baking, quality, gas chromatography-ion mobility chromatography

## Abstract

**Introduction:**

The nutritional value of duck meat is well acknowledged due to its low cholesterol and high protein content. Nevertheless, the impacts of deep-frying and baking on its quality characteristics are not extensively documented in literature.

**Methods:**

The objective of this study is to examine the effects of deep-frying, pre-boilingdeep-frying, baking, and pre-boiling-baking on the quality attributes, water distribution, microstructure, and flavor characteristics of duck jerky.

**Results and discussion:**

The findings revealed that the deep-frying group had better quality attributes than the baking, pre-boiling-deep-frying, and pre-boiling-baking groups. The deepfried duck jerky had a higher a* value (4.25) and a lower b* value (5.87), with a more appropriate texture profile, and had the highest comprehensive impression score (5.84). Moreover, the drying rate was faster, and the intensity of the free water and oil signal was significantly elevated in the deep-frying group. The microstructure results indicated that the muscle fibers in the deep-frying group were closely packed, whereas those in the baking group were relatively loose. Furthermore, the GC-IMS test revealed that the deep-fried duck jerky had a wider range of volatile flavor compounds, including 11 unique compounds that were only found in this particular product.

## 1 Introduction

There is an increasing demand for meat products that are high in nutritional value, convenient, and have distinct flavors (1). Due to its high protein, low cholesterol, and low-fat content, duck meat is a premium poultry product that consumers have widely recognized as a wholesome source of animal-based food. Compared to pork and beef, duck is rich in unsaturated fatty acids, like omega-3 and omega-6, which are essential for maintaining cardiovascular health (2). Duck meat provides essential minerals such as potassium, sodium, magnesium, phosphorus, and B-complex vitamins, all of which are crucial for maintaining a healthy energy metabolism and nervous system (3). Shim et al. (4) identified duck meat as a noteworthy protein source that contains essential amino acids, peptides, and antioxidants. Therefore, developing duck meat products with unique nutritional characteristics is of practical importance.

Duck jerky was a type of thermally processed duck meat product. It was a meat product made from fresh duck meat as the main raw material after several procedures, marinating with seasonings and spices, or cooking (5). Due to its characteristics, such as its fluffy and crumbly texture, mild meat quality, and needing no fire, it was gradually accepted by consumers as a convenient occasional food with a stable shelf life (6). The process of cooking duck jerky is an important factor that affects its quality. In the field of food production and processing, it is critical to remove excess water, inhibit microbial growth and endogenous enzyme activity to facilitate preservation, transportation, and marketing, and extend the shelf life of food products while preserving color, aroma, flavor, and shape. The different processing methods used for producing duck jerky can significantly impact its quality parameters, including color, flavor, nutritional value, and flavor compounds, which are crucial attributes related to consumer satisfaction (7). These methods have some drawbacks, such as producing duck jerky with a hard texture that is difficult to chew, so it is critical to improve and develop duck jerky processing methods.

With the growing meat processing industry, various thermal processing techniques (e.g., frying, steaming, grilling, and boiling) were developed to achieve highly nutritious and palatable flavor profiles (8). These methods are the most effective in terms of eliminating microorganisms that cause foodborne diseases (9). Therefore, heat treatment (cooking) is required before meat products can be consumed (10). Preboiling is an auxiliary cooking process used in the thermal processing of duck meat. It is designed to remove off-flavors from the raw meat, change the texture and taste of the meat, shorten the subsequent processing time, and improve the meat quality of the product. The boiling water denatures the proteins on the surface of the raw meat before cooking to form a protective layer, thus reducing the loss of nutrients. Baking and deep-frying are considered to be two of the oldest dry-heat cooking processes (11). Baking is a dry-heat cooking process for raw meat. High-temperature baking causes the meat to undergo the Maillard reaction and lipid oxidation reaction, resulting in the bright red or brownish-red color of the baked meat products and an attractive aroma (12). Baking increases the hardness and chewiness of meat products, and high-temperature baking has a significant effect on the jerky flavor substances (13). Deep-frying is a dry-heat cooking process using fats and oils as a heat exchange medium. During the process, proteins, fatty acids, and microcomponents undergo chemical changes, resulting in a distinct flavor, color, and texture (14). Deep-frying is widely used for chicken meat (15), grass carp fillets (8), and squid (16). Consequently, choosing the appropriate processing methods to meet the needs of consumers who value quality and nutrition is crucial (17). However, comparative research that evaluates the qualitative effects of deep-frying and baking on the quality of duck jerky is limited. Furthermore, the mechanisms that lead to quality differences during the different processing methods need to be elucidated.

As a result, this study focused on the effects of deep-frying and baking on the quality attributes, water distribution, and flavor characteristics of duck jerky and the relationships between these parameters. It also aims to determine the optimal processing method for the processing of duck jerky.

## 2 Materials and methods

### 2.1 Materials and reagents

Cherry Valley ducks were provided by Henan Huaying Agricultural Development Co., Ltd. Seasonings were purchased from a local supermarket. Also, disodium hydrogen phosphate, sodium dihydrogen phosphate, glutaraldehyde, ethanol, isoamyl acetate, and acetone were obtained from Sinopharm Chemical Reagent Co., Ltd. All chemicals and reagents used were of analytical grade.

### 2.2 Processing methods

Frozen duck breasts were microthawed at 4°C before cutting into 7 × 1.5 × 1 cm rectangular meat strips along the muscle fibers. The meat strips were then placed in water for complete thawing and soaking until free of blood and water. They were fished out and drained, and then 1% salt, 1.5% chili powder, 3% cooking wine, 0.75% pepper powder, and 1.25% ginger powder were added to the meat by weight and mixed well. Then, the strips were placed in the refrigerator at 4°C to marinate at low temperature for 12 h and divided into four groups.

#### 2.2.1 Deep-frying

The marinated duck meat was deep-fried in a pan at 170°C for 610 s until the water content reached approximately 28%. The oil was completely drained off before measurement.

#### 2.2.2 Pre-boiling-deep-frying

The strips of marinated duck meat were heated in boiling water until boiling and then drained of surface water. Subsequently, they were placed in a frying pan set at 170°C to dehydrate and cook for 510 s. The strips were deep-fried to reduce the moisture content to around 28%.

#### 2.2.3 Baking

The marinated duck meat was dehydrated in an oven at 170°C for 114 min until the water content was approximately 28%.

#### 2.2.4 Pre-boiling-baking

The pretreatment was the same as that in Section 2.2.2. The duck meat was drained of surface water, then placed in the oven, and baked at 170°C until the water was reduced to about 28%.

### 2.3 Sensory scores

According to Fu et al. (18), a sensory evaluation of duck jerky (acceptability test) was implemented in a group of 10 members. The sensory evaluation was scored on a seven-point hedonic scale: 1 = extremely dislike; 2 = moderately dislike; 3 = slightly dislike; 4 = neither like nor dislike; 5 = slightly like”; 6 = moderately like; 7 = extremely like. The panelists were randomly selected from students and faculty members with expertise in the research group, and the sensory properties of duck jerky were tested in one session. The sensory attributes evaluated were spiciness, numbness, saltiness, greasiness, duck flavor, meat flavor, tenderness, chewiness, elasticity, meat color, tissue status, and overall impression.

### 2.4 Color

The L* (brightness), a* (red-green value), and b* (yellow-blue value) values of the samples were determined using a colorimeter (CR-400; Konica Minolta, Tokyo, Japan). Analyses were performed six times to ensure reproducibility. The color device was calibrated using its white ceramic plate before actual use. The initial values of the white ceramic plate were L* = 94.6, a* = −1.9, and b* = −2.8.

### 2.5 Texture profile analysis (TPA)

The texture properties of the sample were analyzed using a TA-XT Plus texture analyser (Stable Micro Systems, UK) with a cylindrical probe (P/40). The sample was formed into a cube (1 × 1 × 1 cm). The TPA measurement conditions were as follows: pretest speed, 2.0 mm/s; test speed, 1.0 mm/s; posttest speed, 1.0 mm/s; strain, 30%; time, 5.0 s; double compression cycle; and trigger force, 5 g. All measurements were performed at room temperature, and the hardness, springiness, cohesiveness, chewiness, and resilience of the sample were assessed.

### 2.6 Dry basis water content

The sample’s weight was recorded periodically, with the sample being removed, weighed, and noted until a stable weight was reached. Then, the water content of the dry base was computed using the subsequent formula:


$$X=\frac{G-G_{C}}{G_{C}}\text{,}$$

where X represents the water content on a dry basis in grams per gram; G denotes the weight of the wet material at a specific weight in grams; Gc is the weight of the completely dry material in the wet material in grams. The weight per unit mass, when dried to a constant weight, serves as the absolute dry material value, Gc.

### 2.7 Low-field nuclear magnetic resonance (LF-NMR)

The duck jerky was cooled and placed in a desiccator for 2 h to balance the water. The samples were measured using an LF-NMR analyser (NMI20-040 V-I, Suzhou Niumag Analytical Instrument Co., Ltd., Suzhou, China) with a proton resonance of 20 MHz at 32°C and a magnetic field strength of 0.5 Tesla. The decay signal of CPMG sequence was collected by a 40 mm diameter radio frequency coil. The parameters were set as follows: 90° pulse width (P1) = 7.52 μs, 180° pulse width (P2) = 14.48 μs, waiting time (TW) = 2,500 ms, number of echoes (NECH) = 10,000, and number of scans (NS) = 8. Then, the transverse relaxation (T2) signals were obtained, including the relaxation amplitude and the time constant during relaxation.

### 2.8 Hydrogen proton density imaging

Magnetic resonance imaging (MRI) analysis was performed using an NMR analyser (NMI20-040 V-I, Suzhou Niumag Analytical Instrument Co., Ltd., Suzhou, China). The sample was placed in the center of the radiofrequency coil of the magnet box and was imaged by MRI using a multilayer spin-echo (SE) sequence.

### 2.9 Scanning electron microscope (SEM)

Sample processing was performed as described by Wang et al. (19), with some modifications. The sample was shaped into a cube (10 × 10 × 5 mm), fixed with 2.5% glutaraldehyde (pH = 7.0) for 24 h, and then rinsed three times with 0.1 mM phosphate buffer (pH = 7.0) for 15 min each time. A series of ethanol (30, 50, 70, 90, 100, and 100%) was used for dehydration (15 min/step). The sample was washed successively in a mixture of alcohol and isopentyl acetate (v: v = 1:1) before being immersed twice in pure isopentyl acetate for 10 min each time. The microstructure of the samples was observed using a scanning electron microscope (Quanta-250FEG, FEI Company, America) at an accelerating voltage of 5 kV. The magnification was set at 200× for all samples.

### 2.10 Volatile flavor compounds (VFCs)

Volatile flavor compounds (VFCs) in the sample were detected using a GC-IMS flavour analyser (FlavourSpec, G.A.S., Dortmund, Germany) equipped with a chromatographic column. The sample (2 g) was placed in a 20 mL headspace bottle and then stirred for 15 min at 60°C. A headspace sample was removed and injected into the GC column (injection needle temperature at 80°C). The GC conditions were as follows: temperature, 60°C; carrier gas, nitrogen (purity ≥99.999%); flow rate, 5 mL/min for 3 min, went up to 50 mL/min in 8 min, then ramped to 150 mL/min within 5 min for 3 min, total run time 19 min. IMS conditions were as follows: drift tube temperature, 45°C; drift gas, nitrogen (≥ 99.999%); flow rate, 150 mL/min.

### 2.11 Statistical analyses

Experiments were performed in triplicate (unless otherwise stated) for each sample. Data were evaluated using one-way analysis of variance (ANOVA) followed by Duncan’s multiple range test using SPSS 24.0 statistical software (SPSS Inc., Chicago, IL, USA) for statistical significance. The results were reported as mean values ± standard deviations, and differences were considered significant when *p* < 0.05. Graphs were generated using Origin 2018.

## 3 Results

### 3.1 Effect of different processing methods on the quality attributes of duck jerky

#### 3.1.1 Sensory scores

**Figure 1:**
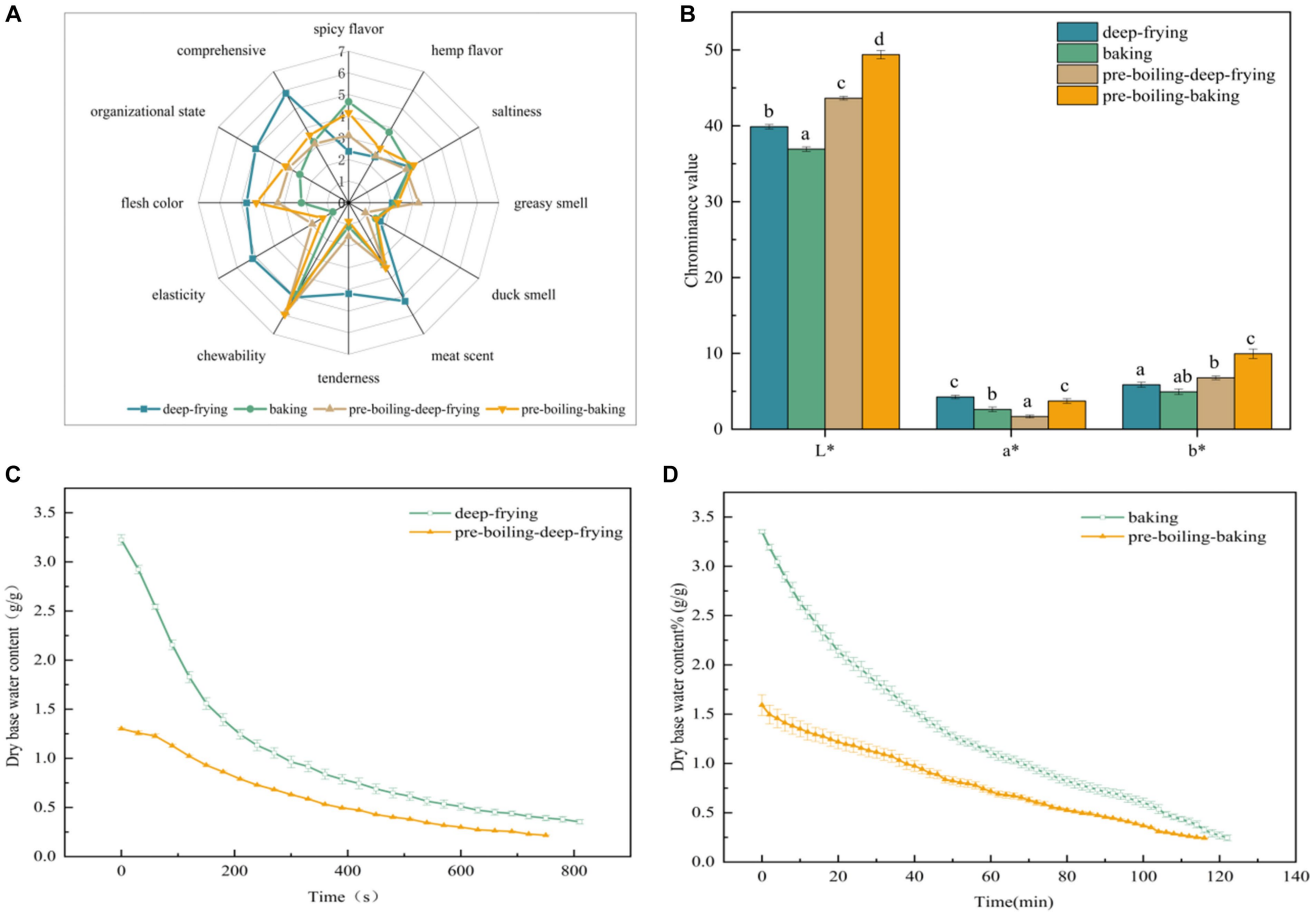
Sensory evaluation **(A)**, color **(B)**, and dry basis water content [**(C)** deep-frying and pre-boiling-deep-frying; **(D)** baking and pre-boiling-baking] of duck jerky produced using different processing methods. Different lowercase letters indicate significant differences between the experimental groups (*p* < 0.05).

Figure 1A depicts the effects of different processing methods on the sensory quality of duck jerky. These processing methods significantly affected (*p* < 0.05) all sensory qualities of the duck jerky. According to the color scores, the deep-fried duck jerky had the best flesh color, with a score of 4.75, whereas the baked jerky had the worst score of 2.2. During the deep-frying process, the color of the duck jerky gradually changed from light red to dark red. During the post-baking processing of duck jerky, a browning reaction occurs, which darkens the product and in turn severely affects its color score. In terms of flavor-related scores, the deep-fried duck jerky scores for meat scent were high. Conversely, the preboiling process may have led to the loss of some flavor compounds in the duck meat. However, the deep-fried jerky effectively preserved the authentic flavor of the duck meat while eliminating the fishy. Regarding duck meat texture, the overall texture of the deep-fried duck jerky was better than that of the other groups. The baked product scored lower; it had a dry and hard texture and lower sensory acceptability. Collectively, the duck jerky subjected to deep-frying treatment was well-received by the general public in terms of meat scent, tenderness, chewability, elasticity, flesh color, organizational state, and composite score compared to other methods. These results were similar to those of Wang et al. (20) in their flavor studies on scallops.

#### 3.1.2 Color

Color is one of the important indicators to evaluate the quality of meat products. The color characteristics of meat products were mainly measured by L* (brightness), a* (redness), and b* (yellowness) values. Figure 1B illustrates the effects of different processing methods on the L*, a*, and b* values of duck jerky. The four processing methods had a significant impact (*p* < 0.05) on the color quality of duck jerky. The L* value of the duck jerky in the pre-boiling-baking group was the highest (49.38), followed by the duck jerky in the pre-boiling-deep-frying group. After preboiling, the working time was relatively shorter, and the time of fat oxidation and Maillard reaction was shortened. Rendalai Si et al. (10) examined the effect of different processes on the nutritional quality of Bactrian camel meat and reached similar conclusions. After 20 min of steaming, L* increased significantly (*p* < 0.05), and the L* value increased and then decreased with increasing heating time. The deep-fried duck jerky had a* value of 4.25, which was significantly higher than that of the pre-boiling-deep-frying and baking groups. Additionally, the b* value of the deep-fried duck jerky reached 5.87, which was substantially lower than that of the pre-boiling-deep-frying and pre-boiling-baking groups (*p* < 0.05). This may be due to the combined effect of the Maillard reaction, caramelization reaction, and some other reactions that occurred in the unboiled duck meat during the deep-frying process, which resulted in a more attractive red color after deep-frying.

#### 3.1.3 TPA

TPA was a deformation test designed to simulate chewing in the human mouth. The TPA parameters offered additional information on the textural characteristics of the meat products (21). Table 1 lists the textural properties of duck jerky under different processing methods. The table shows that the processing methods significantly affected the hardness, springiness, and chewiness of duck jerky (*p* < 0.05), particularly the hardness and chewiness (*p* < 0.001). The hardness level of duck jerky subjected to different processing methods can be arranged in the following sequence: pre-boiling-baking > baking > pre-boiling-deep-frying > deep-frying. This finding indicates that there could be a possible correlation with the thermal contraction of the myofibrillar protein, leading to the formation of a denser muscle fiber structure and ultimately improving the overall muscle fiber structure. Wang et al. (20) reached similar conclusions in their study on the effects of different cooking methods on the texture of scallop muscle, with samples from the baking group being harder and more chewy. The springiness of the duck jerky obtained after preboiling was significantly higher than that of the non-preboiled jerky (*p* < 0.05). This could be attributed to the proteins in the duck meat undergoing denaturation and coagulation after preboiling, leading to a cross-linking of large macromolecules. As a result, the springiness of the duck jerky is increased. The four processing methods did not have a significant effect on the cohesiveness of the duck jerky (*p* > 0.05). The sensory scores confirmed that the deep-fried duck jerky displayed superior textural properties to those of the other treatment groups.

**Table 1:** Texture profile analysis of duck jerky under different processing methods.

	Deep-frying	Baking	Pre-boiling-deep-frying	Pre-boiling-baking
Hardness/g	2864.21 ± 188.61^a^	7528.05 ± 642.25^c^	4349.52 ± 367.20^b^	9170.30 ± 157.90^d^
Springiness	0.88 ± 0.003^b^	0.84 ± 0.02^a^	0.92 ± 0.003^bc^	0.93 ± 0.009^c^
Cohesiveness	0.79 ± 0.004^a^	0.75 ± 0.05^a^	0.73 ± 0.01^a^	0.82 ± 0.01^a^
Chewiness	2667.67 ± 278.97^a^	3129.77 ± 87.11^ab^	3399.34 ± 136.53^b^	5201.99 ± 157.61^c^

Different lowercase letters indicate significant differences in the qualitative properties of each experimental group (*p* < 0.05).

### 3.2 Effect of different processing methods on the water of duck jerky

#### 3.2.1 Dry basis water content

Figures 1C,D demonstrate the dry basis water content of duck jerky obtained by the four processing methods. This was due to the higher water content of duck meat in the early drying stage, which resulted in the absorption of more heat energy in the early deep-frying or baking stage, triggering a fast evaporation and drying rate. During the middle and later stages of dehydration, a rigid exterior layer forms on the meat surface. This causes a gradual decrease in the water diffusion rate from the product’s interior to the surface, which is lower than the evaporation rate on the surface. Consequently, the dehydration process slows down over time. As shown in the figure, the drying rate decreased significantly in the later stages of deep-frying. The muscle fibers in the duck meat became substantially taut as a result of the high temperature used in deep-frying, impeding the spread of moisture from the food. For the same water content, deep-frying took significantly less time than baking. Juarez et al. (22) demonstrated that different processing methods resulted in water loss and that deep-frying resulted in the fastest water reduction.

#### 3.2.2 LF-NMR

**Figure 2:**
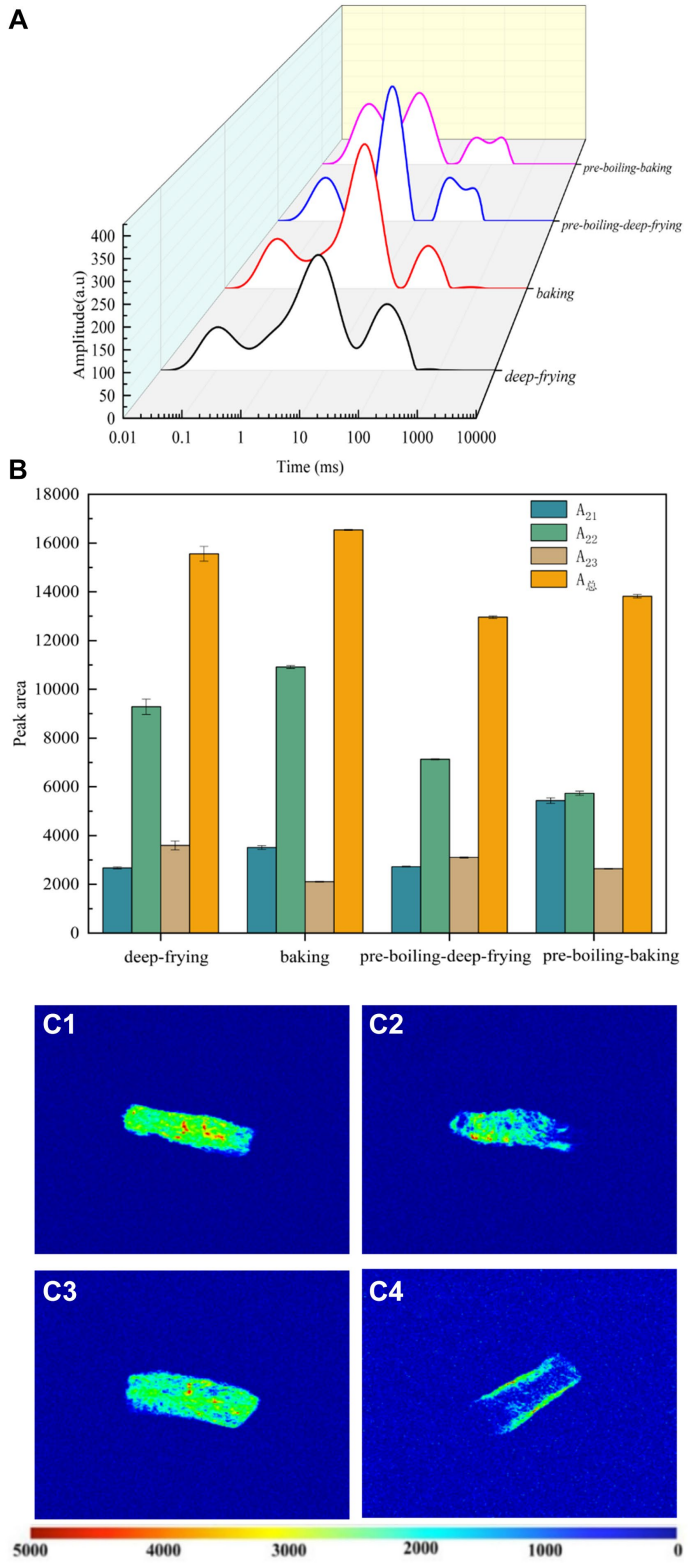
T_2_ relaxation spectra **(A)**, peak area change of water **(B)**, and NMR images [**(C1)** deep-frying; **(C2)** baking; **(C3)** pre-boiling-deep-frying; **(C4)** pre-boiling-baking] of duck jerky from different processing methods. Different lowercase letters indicate significant differences between groups (*p* < 0.05).

Water content and water status are closely related to meat texture. Water in jerky was classified into three types: bound water, immobilized water, and free water. The length of the relaxation time T_2_ was known from the NMR principle to vary with the chemical environment in which the proton was located, as did the degree of freedom of the water (23). The water was bound more tightly to the compound when the relaxation time T_2_ was shorter, making it less easily released. However, when the proton had more freedom, it was easier to release the water. Thus, the relaxation time T_2_ can be used to understand the migration law of water during the different processing methods of duck jerky, reflecting the dynamic properties of water, such as diffusion and state (24). The different processing methods had a highly significant effect on the transverse relaxation time T_2_ of duck jerky (*p* < 0.01) (Figure 2A). Three main relaxation peaks can be summarized in the transverse relaxation curve, namely, T_21_ (< 0.5 ms), T_22_ (0.5–20 ms), and T_23_ (20–1,000 ms). The T_21_ component, which had the shortest relaxation time, can be seen as the most tightly bound water. The degree of water freedom of the T_22_ component of the relaxation time was intermediate between bound and free water and was susceptible to transformation, defined as immobilized water. It was the main form of water present in the muscle as water bound to the surface of the sample and inside the myogenic fibers by molecular adsorption. The water of the longest relaxation time component, T_23_, had the mobility of water in an aqueous solution and was therefore defined as free water. It had little binding to the internal dry matter of the food and was primarily found outside the myogenic fibrous protein or in the interstitial spaces of the myocytes. This fraction of water was highly mobile and readily available to microorganisms and enzymes and was the most likely water to be lost during the dehydration process (25). Similar water attributes were also reported for beef (26), surf calm (27), and instant sea cucumber (28).

The signal intensity was defined as the hydrogen proton density of the corresponding water or fat (29). Figure 2B demonstrates the peak areas corresponding to each water, which were counted in order to understand the effects of the different processing methods on the various water states in the duck meat. The T_21_ signal intensity of the pre-boiling-baking duck jerky was significantly higher than that of the other treatment groups (*p* < 0.05). In comparison, the T_22_ signal intensity was significantly lower than that of the other treatment groups (*p* < 0.05). This may be due to the contraction of the myofibrillar network structure after extended baking of pre-boiled duck jerky, causing the conversion of relatively immobile water inside the myogenic fibers into bound water. Song et al. (30) reported that less free water indicated the change of water within the structure of myofibrils in meat. Muscle tissue was inhomogeneous, and T_21_ was positively correlated with the cross-sectional area of myofibrils and the length of the muscle nodes. A decrease in this value indicated a reduction in the mobility of the immobile water. Most of the water in duck meat was immobile water T_22_, and the signal intensity of T_22_ relaxation in deep-fried and baked duck jerky was significantly higher than that in duck jerky obtained by preboiling (*p* < 0.05). This may be due to changes in the spatial conformation of myofibrillar proteins in duck jerky during pre-boiling. The water content of the duck jerky and its attributes and state of presence were affected during the cooking process. The signal intensities of free water and fat T23 were significantly higher in the deep-fried duck jerky than those in the other treatment groups (*p* < 0.05). The increased free water content in the deep-frying cohort led to better textural and sensory attributes for the deep-fried duck jerky. This was particularly evident in hardness and chewiness, which were corroborated by the above-mentioned textural and sensory findings. However, whether the signal value of T_23_ also included the fat and deep-frying oil content of the duck jerky warrants further investigation.

#### 3.2.3 Hydrogen proton density

Changes in H^+^ proton density images of duck jerky during the different processes were observed using MRI imaging. MRI can visualize the migration and properties of water in food and detect the changes that occur during different processes (31). Figures 2C1–C4 demonstrate the corresponding pseudocolor images, where red indicates high proton signal density, and blue indicates low proton signal density (32). The water attributes of deep-fried duck jerky were more uniform, and the internal organization of duck jerky by pre-boiling-baking was extremely tight. The cohesiveness and chewiness of duck jerky produced by pre-boiling-baking were higher than those in the other treatment groups, resulting in poorer textural characteristics of the samples. This corresponded to the findings of the study on the effect of different processing methods on the texture of duck jerky. It can be inferred that the deep-frying process produced a much more uniform distribution of water in the duck jerky, leading to superior textural and sensory characteristics.

### 3.3 Effect of different processing methods on the microstructure of duck jerky

**Figure 3:**
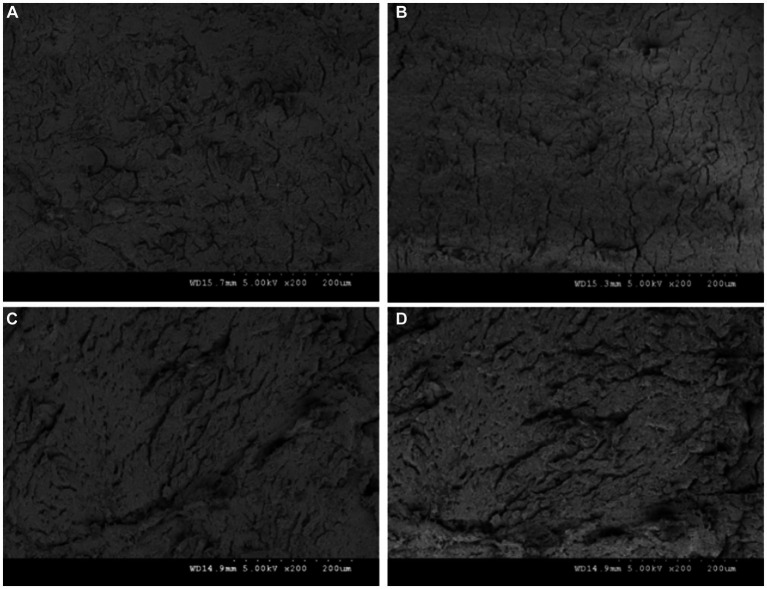
Microstructure of duck jerky produced using different processing methods. **(A)** Deep-frying; **(B)** baking; **(C)** pre-boiling-deep-frying; **(D)** pre-boiling-baking.

Figure 3 illustrates the microstructure of duck jerky obtained by different processing methods observed using scanning electron microscopy. The muscle fibers of deep-fried duck jerky were tightly arranged with almost no gaps. However, those of the baked duck jerky appeared loose with significant gaps because the high temperatures during the prolonged baking and heating process caused the myofibril structure to lose its integrity and the myofascicles to contract, destroying its compact structure. It was previously reported that heating causes denaturation of myosin and the proteins that make up the myofibril membrane, which reduces the binding force of myogenic fibers to water and increases myofibril contraction, leading to the destruction of the myogenic fiber structure (24). Preboiling increased the contraction level in duck muscle fibers, resulting in irregularly textured muscle cross-sections. Moreover, the muscle tissue structure suffered disruption, and the muscle structure became lax, leading to the formation of smaller gaps and significant fractures in the myofibril bundles. Qi et al. (23) suggested that the high temperature irreversibly changed the meat’s microstructure. In contrast, deep-frying caused less damage to the muscle fiber structure in the duck jerky compared to the other methods, resulting in a more condensed and intact muscle fiber bundle structure. As a result, the qualitative properties of the deep-fried duck jerky were found to be better.

### 3.4 Effect of different processing methods on volatile flavor compounds of duck jerky

#### 3.4.1 Differential analysis of two-dimensional profiles based on GC-IMS

**Figure 4:**
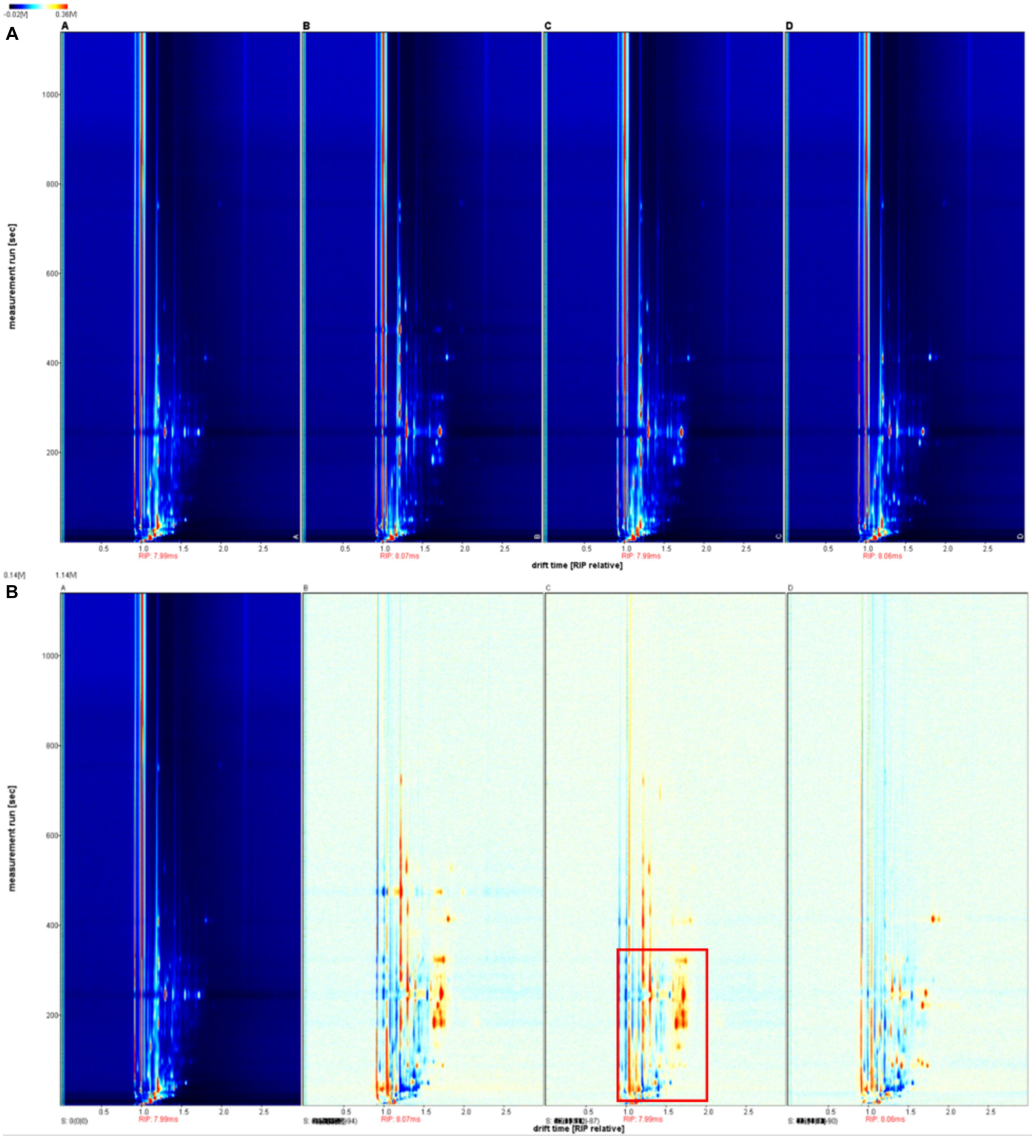
GC-IMS two-dimensional spectra **(A)** and comparison of VOCs **(B)** of duck jerky produced using different processing methods. A, Baking; B, pre-boiling-baking; C, deep-frying; D, pre-boiling-deep-frying.

Figure 4A demonstrates the two-dimensional GC-IMS profiles of duck jerky VFCs obtained by different processing methods. The y-axis was the retention time of the gas chromatograph, while the x-axis was the relative ion drift time (33). Each data point represented a VFC, and its relative intensity was indicated by color, with darker red corresponding to higher concentrations and darker blue corresponding to lower concentrations (33). The changes in the types and concentrations of VFCs in duck jerky under different processing methods over time were visualized using gas phase ion mobility spectrograms.

Figure 4B displays the variations in VFCs among the four processing methods that were compared using the difference comparison software, with the baking samples serving as a benchmark. The red parts of the plots for pre-boiling-baking and deep-frying were significantly more abundant than those for baking. Moreover, the deep-frying process was the most significant, indicating that the deep-fried duck jerky was the richest in VFCs. The GC-IMS profiles of the samples obtained from the four processing methods exhibited significant variations, all falling within the retention time range of 0–380 s (shown in the red box in the figure). This signal component exhibited a short retention time and a high macromolecular weight, which strongly affected the sensory quality of the products. Consequently, alterations in water content due to processing methods significantly influenced the VFCs of the product.

#### 3.4.2 Fingerprint analysis based on GC-IMS

**Figure 5:**
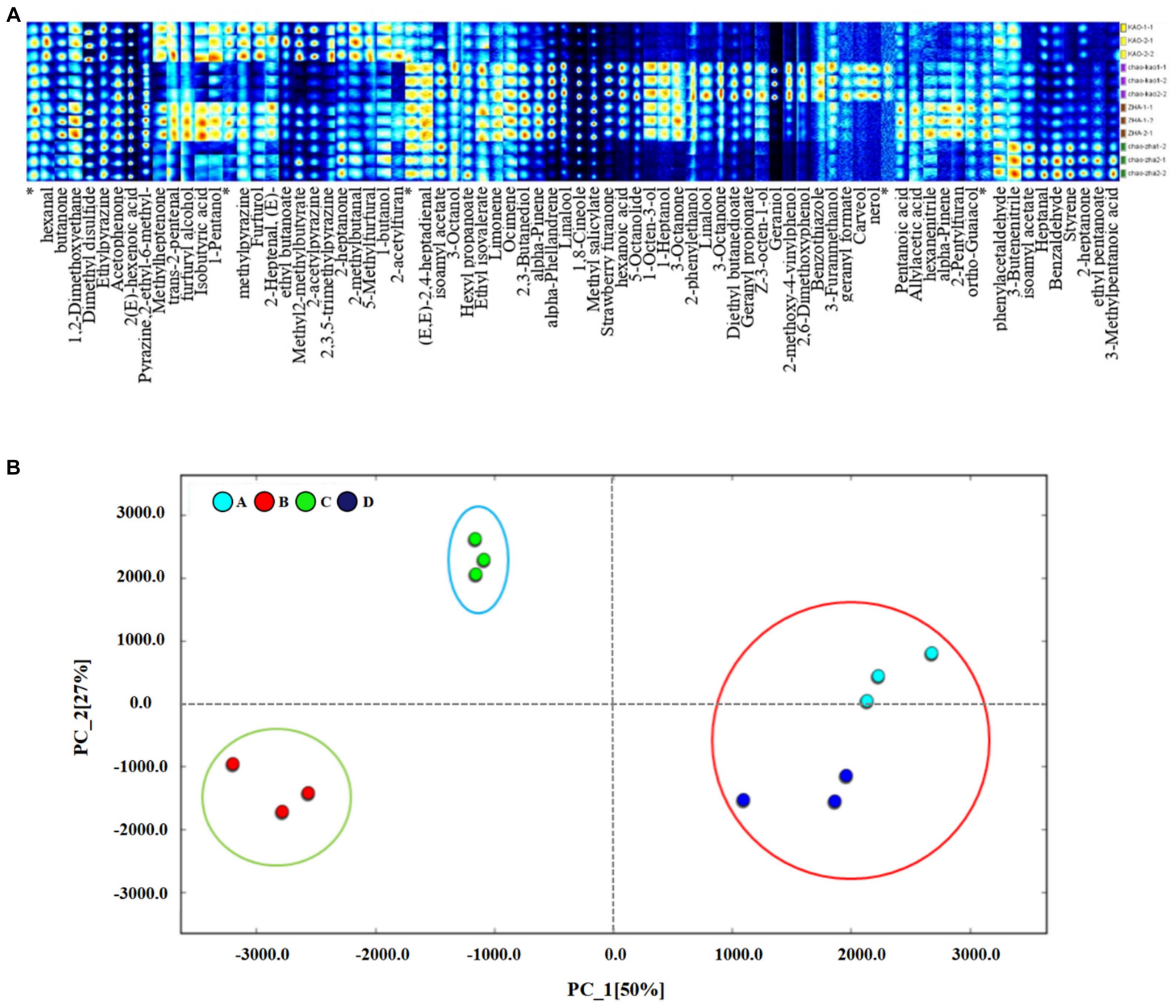
Characteristic flavor fingerprint **(A)** and PCA analysis **(B)** of characteristic flavor fingerprints of duck jerky produced using different processing methods. A, baking; B, pre-boiling-baking; C, deep-frying; D, pre-boiling-deep-frying.

To elucidate the dissimilarities in particular flavor compounds found in duck jerky subjected to distinct processing techniques, we amalgamated the characteristic flavors compounds after integration and characterization. Then, we selected all peaks for fingerprint comparison. The differences in volatile organic compounds (VOCs) between samples could be clearly distinguished by fingerprint comparison as Xiao et al. (34) found significant differences in the flavor components of different edible parts of carp. In Figure 5A and Supplementary Table S1, 78 volatile compounds were detected in the duck jerky treated by the four methods. After qualitative analysis, according to the database, 73 VFCs were detected qualitatively, including eight ketones, 10 aldehydes, 12 esters, and 18 alcohols. The horizontal coordinates in Figure 5A were the different volatile components, and the vertical coordinates were the names of the different samples, from top to bottom, baking, pre-boiling-baking, deep-frying, and pre-boiling-deep-frying, respectively. Each row of the fingerprint map represented all the signal peaks detected for that sample, and each column described the signal peaks of the same VFCs in different samples. The brighter the color, the higher the content.

Compounds in the A region of the graph were detected in all four processing methods and collectively displayed roasted meat, fruit, caramel, and nut flavors. Preboiling caused the duck jerky to lose some nutrients as well as some VFCs, as shown in Zone B. Among these, methylheptenone with a fresh fruit aroma was more pronounced in the deep-fried duck jerky. This could be due to the oxidation of fatty acids and degradation of amino acids caused by high-temperature heating. During the process of high-temperature baking, duck meat undergoes fat oxidation and the Maillard reaction. Consequently, baking aroma compounds are produced, which are specific to Zones C and E. Nine of the compounds were found only in the baked samples. Moreover, long-term high-temperature baking causes the protein fat in the meat to degrade and react with one another, resulting in various flavor compounds.

VFCs were most abundant in deep-fried duck jerky, with a total of 50 volatile components detected, 11 of which were only found in deep-fried duck jerk. The threshold for aldehydes was lower, and the higher its proportion in the meat, the greater the contribution to the flavor of duck jerky (35). Esters were generated by the esterification reaction between alcohols and acids, resulting in a fruity flavor with a lower threshold to mask the off-flavor (36). The Maillard reaction, thermal degradation of amino acids, and thermal degradation of thiamine usually produced heterocyclic compounds such as pyrazines and furans. A low threshold is beneficial for the good flavor of the product. The formation of furan compounds, 2-pentylfuran, was the oxidation product of linoleic acid, which had a bean, fruit, and vegetable aroma. The amount of hydrocarbons present is significantly high; however, hydrocarbons possess a weak ability to affect the taste of the product, thus having a minor influence on the overall flavor. Nevertheless, the deep-fried duck meat samples contained a relatively high concentration of hydrocarbon compounds, which proved to be useful in enhancing the flavor of duck jerky.

#### 3.4.3 Principal component analysis (PCA)

The PCA plot provides a visual representation of the variations between different samples. Proximity between samples indicates minor differences, whereas distant samples depict significant differences. According to the obtained ion mobility fingerprint results, PCA analysis was conducted on duck jerky samples that underwent various processing methods, and the outcomes are displayed in Figure 5B. The contributions of PC1 and PC2 were 45 and 26%, respectively, while the cumulative contribution reached 71%. The data suggest that the majority of VFC information in the duck jerky samples, which were processed using different methods, can be attributed to the overall contribution. Moreover, there was a noticeable disparity in the flavors of the samples obtained through various processing methods, while the samples within the same group were relatively concentrated in their distribution. This indicated the good parallelism of the samples and the good differentiation effect of GC-IMS on the flavor compounds of duck jerky from different processing methods. The baking group and the pre-boiling-deep-frying group were relatively close to each other. This implies, to a certain degree, that the resulting taste characteristics were comparable for both processing techniques.

## 4 Conclusion

The four drying methods had significant effects (*p* < 0.05) on the sensory scores, color, and texture of the duck jerky. The preboiling process led to a severe contraction of the muscle fibers of the duck jerky and sustained substantial damage to its muscle fiber structure. Conversely, the muscle fibers of the baked duck jerky were relatively loose, exhibiting significant gaps, whereas those of the deep-fried duck jerky maintained their intact structure and good alignment. Further analysis indicated that the deep-fried duck jerky had a faster drying time and a more uniform distribution of water, with elevated levels of free water and fat content (*p* < 0.05). Furthermore, the deep-fried duck jerky displayed the largest variety of VFCs, with 50 VFCs identified. Overall, this study establish a theoretical foundation for the advancement of duck products or meat products processed thermally.

## Data availability statement

The original contributions presented in the study are included in the article/Supplementary material, further inquiries can be directed to the corresponding author.

## Author contributions

YP: Methodology, Writing – original draft. XG: Data curation, Writing – original draft. XS: Validation, Writing – original draft. YH: Writing – review & editing. YA: Funding acquisition, Writing – review & editing. HW: Writing – review & editing. WH: Funding acquisition, Supervision, Writing – review & editing.
