# Supplementary figures and images for: Effects of deep frying and baking on the quality attributes, water distribution, and flavor characteristics of duck jerky
Source: Front Nutr. 2024 Feb 7;11:1309924. doi: 10.3389/fnut.2024.1309924 (PMC10882714; doi:10.3389/fnut.2024.1309924)

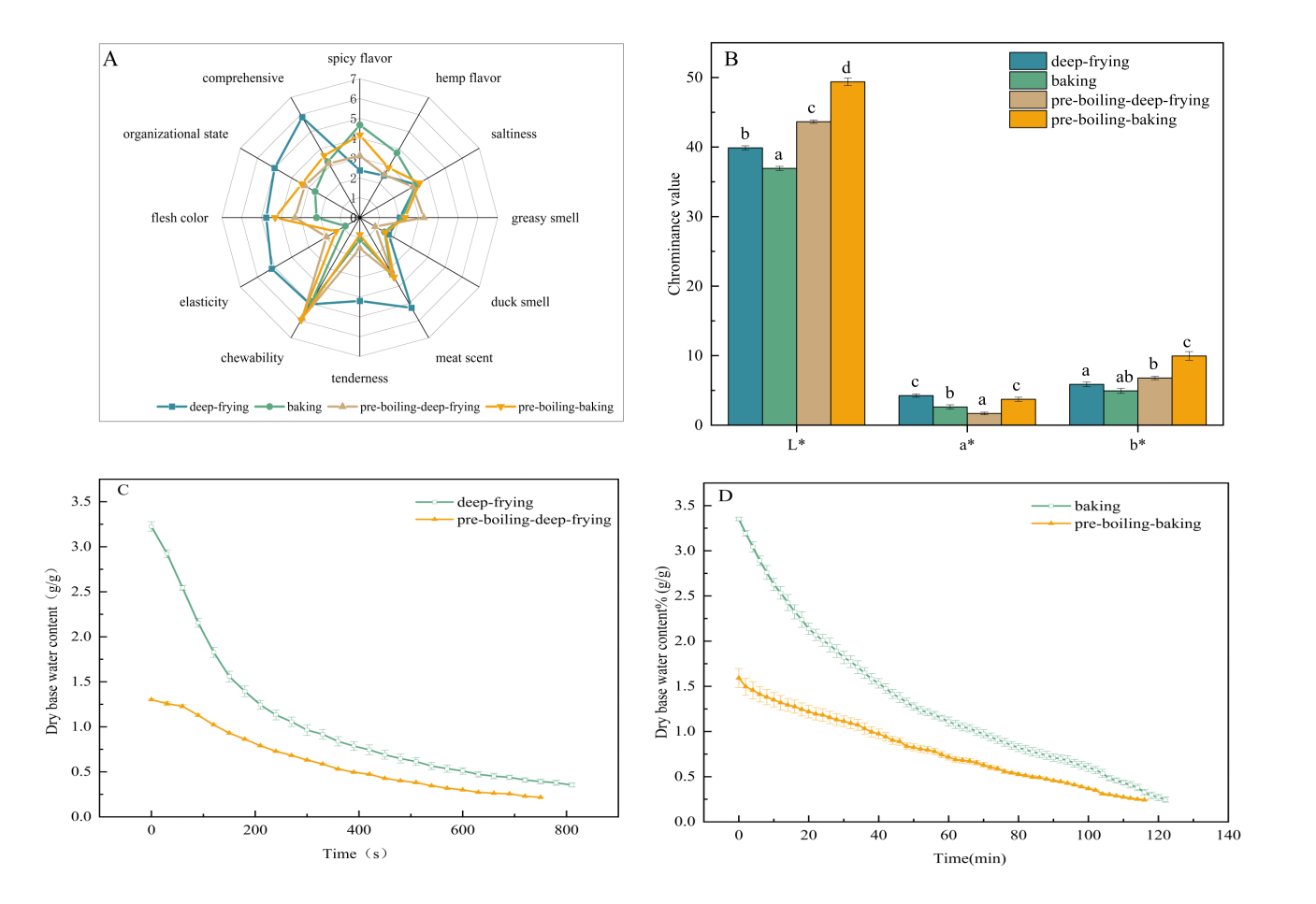

Supplement: Supplementary file 1 [file Data_Sheet_1.ZIP › Supplementary Figures/Figure1.png]

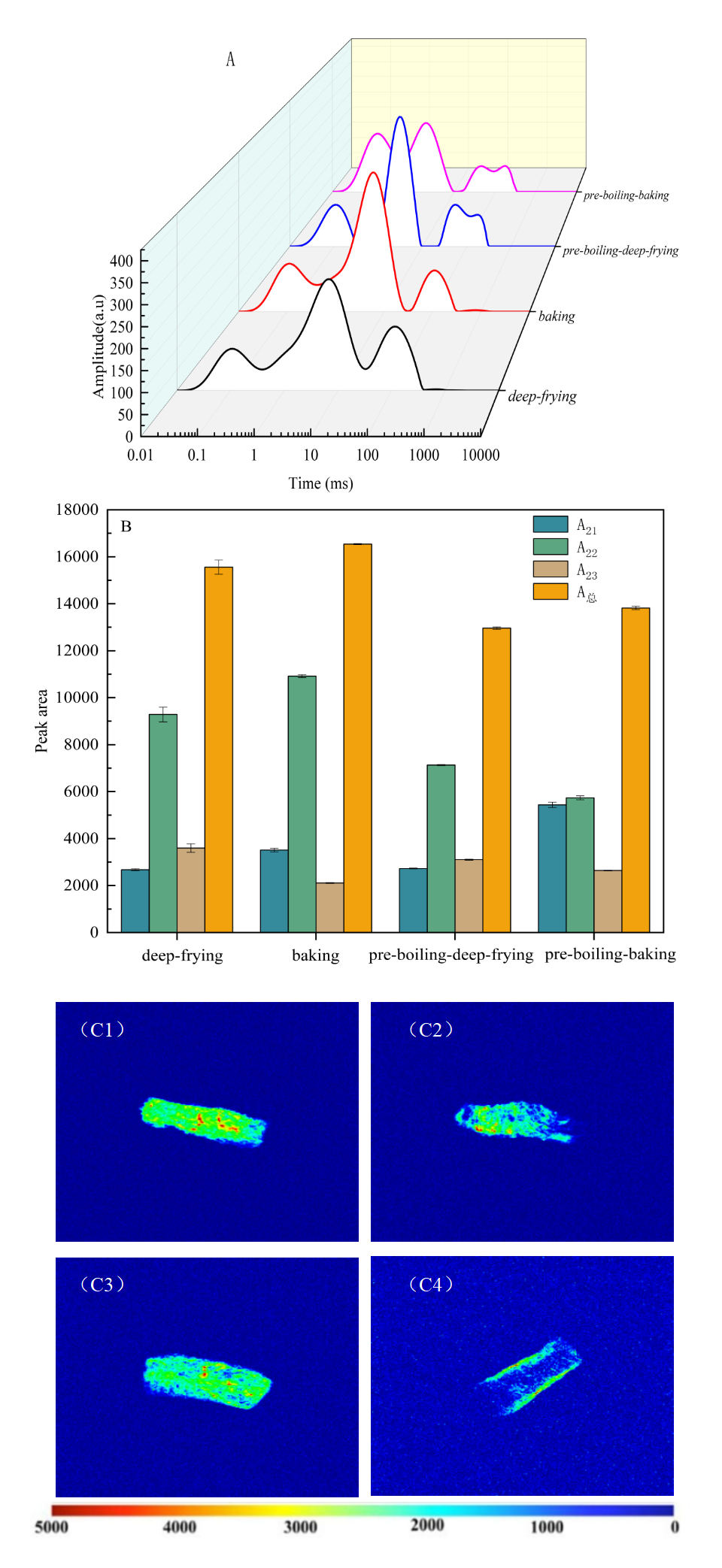

Supplement: Supplementary file 1 [file Data_Sheet_1.ZIP › Supplementary Figures/Figure2.png]

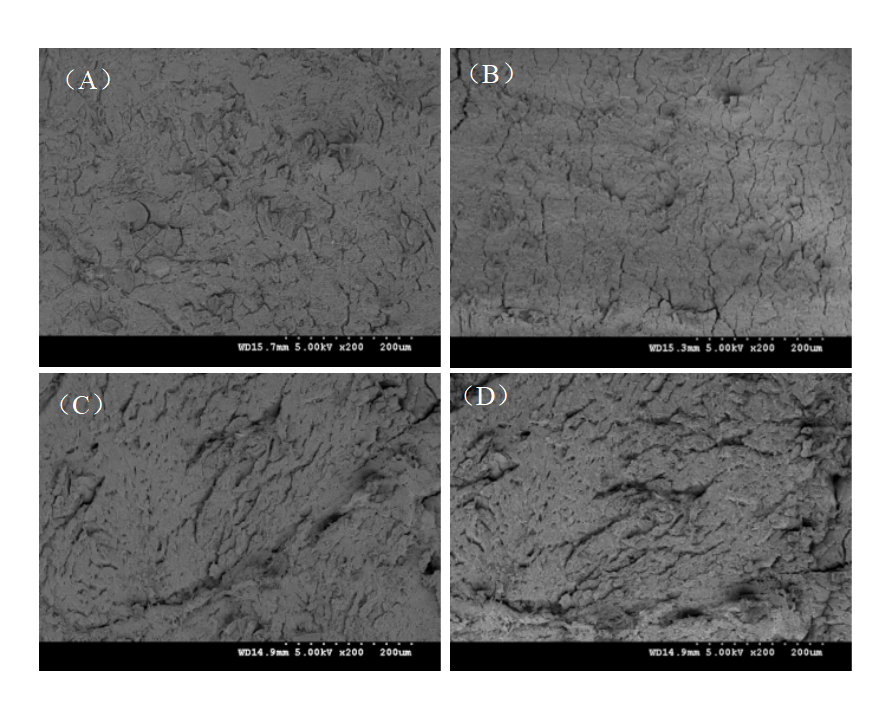

Supplement: Supplementary file 1 [file Data_Sheet_1.ZIP › Supplementary Figures/Figure3.png]

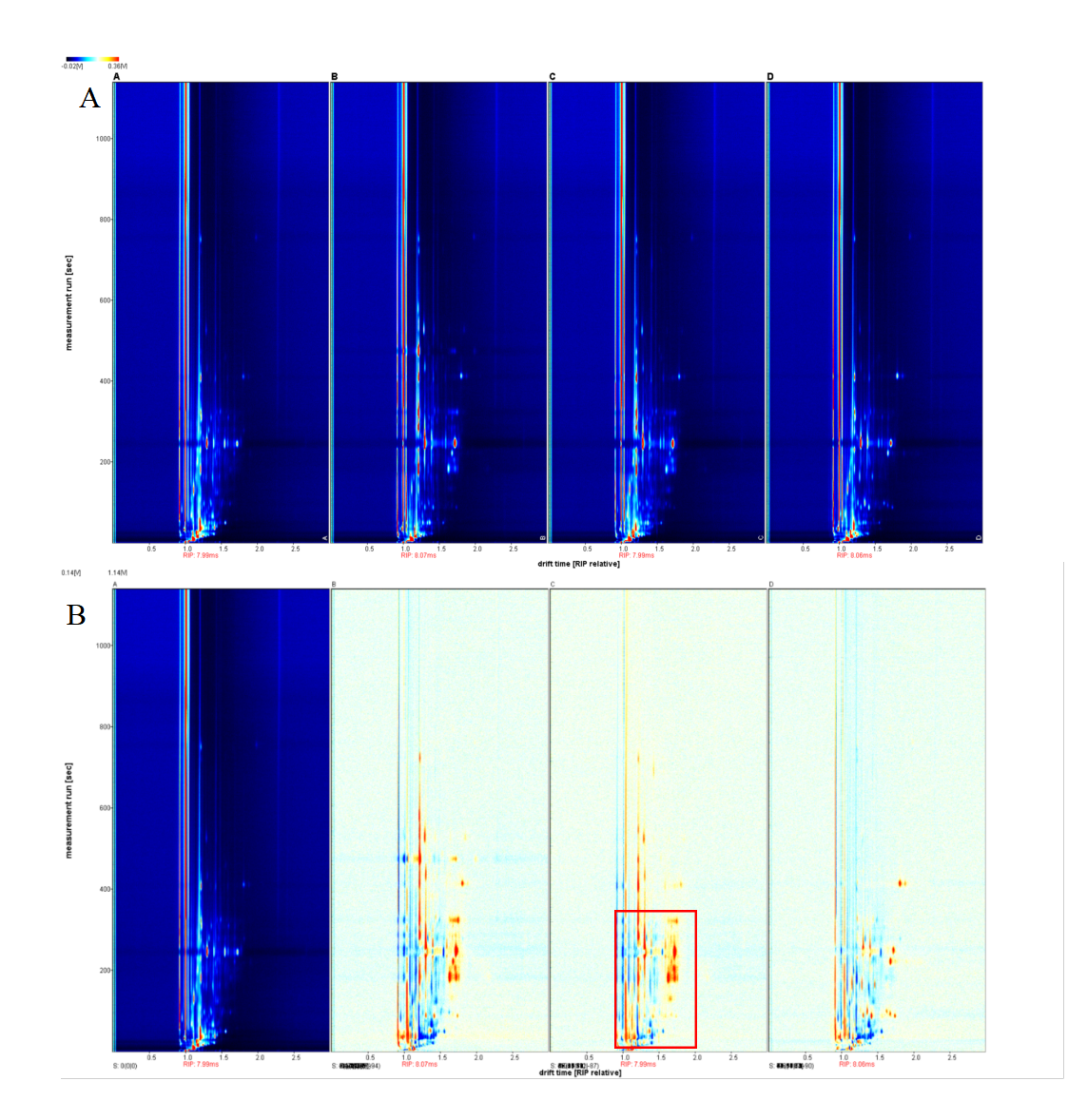

Supplement: Supplementary file 1 [file Data_Sheet_1.ZIP › Supplementary Figures/Figure4.png]

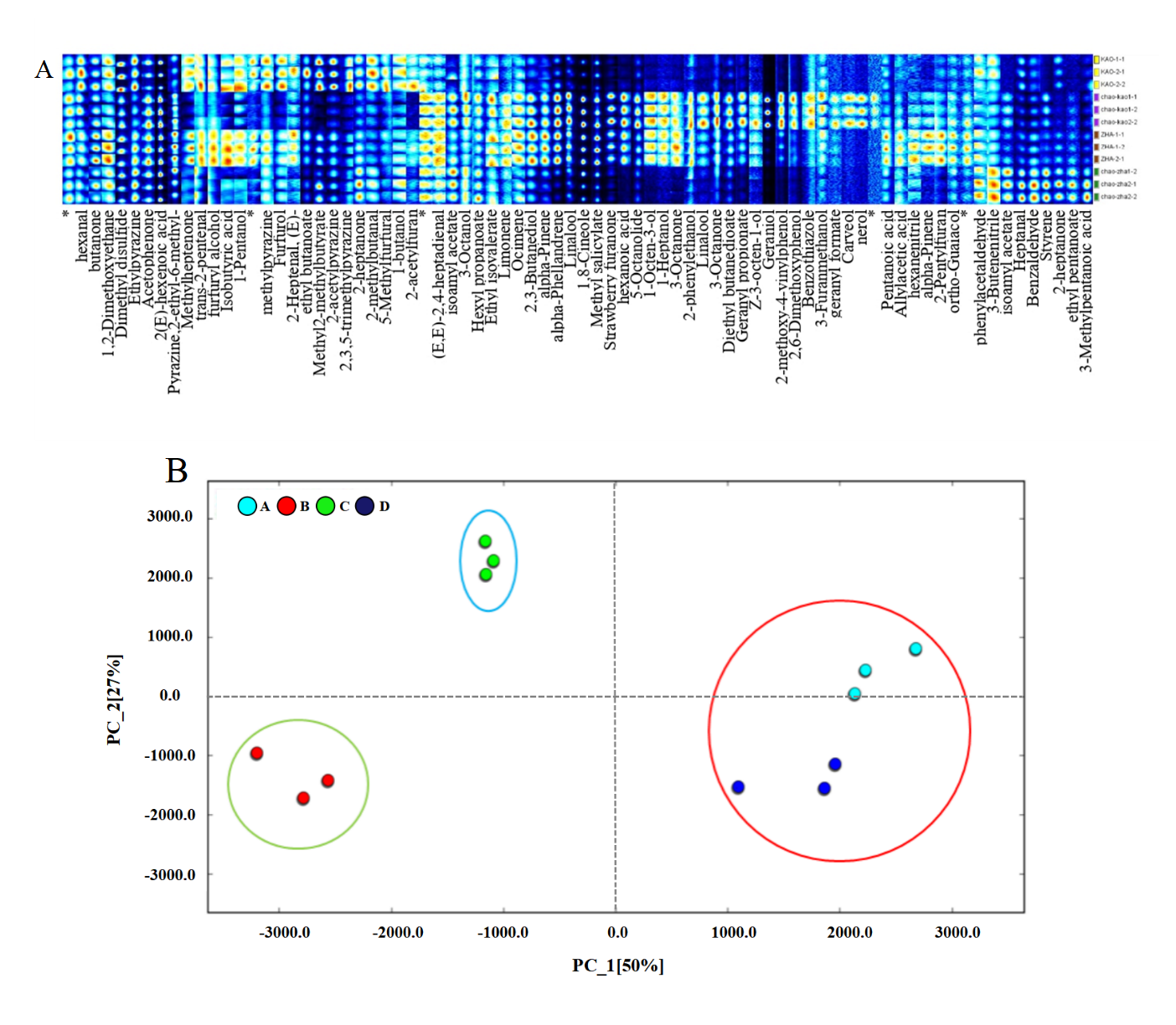

Supplement: Supplementary file 1 [file Data_Sheet_1.ZIP › Supplementary Figures/Figure5.png]
