# Supplementary material for: Effects of deep frying and baking on the quality attributes, water distribution, and flavor characteristics of duck jerky
Source: Front Nutr. 2024 Feb 7;11:1309924. doi: 10.3389/fnut.2024.1309924 (PMC10882714; doi:10.3389/fnut.2024.1309924)
Supplement: Supplementary file 2 [file Table_1.DOCX]

Supplementary Material

# Supplementary Tables

**Supplementary Table 1 Volatile flavor compounds of duck jerky with different processing methods**

| **number** | **Compounds** | **Aroma characteristic** | **CAS** | **Molecular formula** | **Retention index** | **Retention time/s** | **Drift time/ms** |
| --- | --- | --- | --- | --- | --- | --- | --- |
| **1** | **Benzaldehyde** | **Aroma of cherry and nut** | **C100527** | **C_7_H_6_O** | **956.5** | **162.271** | **1.1468** |
| **2** | **Methylheptenone** | **With fruit aroma, fresh fragrance** | **C110930** | **C_8_H_14_O** | **991.1** | **201.985** | **1.1654** |
| **3** | **Linalool** | **It has lily of the valley fragrance** | **C78706** | **C_10_H_18_O** | **1099.9** | **320.672** | **1.2145** |
| **4** | **Furfurol** | **Special smell similar to benzaldehyde** | **C98011** | **C_5_H_4_O_2_** | **842.6** | **69.387** | **1.0819** |
| **5** | **methylpyrazine** | **It has nut fragrance, mildew fragrance, roast fragrance and soil fragrance** | **C109080** | **C_5_H_6_N_2_** | **843.3** | **69.72** | **1.0966** |
| **6** | **trans-2-pentenal** |  | **C1576870** | **C_5_H_8_O** | **760.3** | **40.397** | **1.1142** |
| **7** | **furfuryl alcohol** | **bitter** | **C98000** | **C_5_H_6_O_2_** | **813.4** | **57.334** | **1.1142** |
| **8** | **ethylpyrazine** | **It has nut fragrance, wood fragrance, soil fragrance, roast fragrance and meat fragrance** | **C13925003** | **C_6_H_8_N_2_** | **931** | **134.718** | **1.1173** |
| **9** | **Methyl2-methylbutyrate** | **With ether fragrance, fruit fragrance, green fragrance** | **C868575** | **C_6_H_12_O_2_** | **754.5** | **38.77** | **1.2042** |
| **10** | **Isobutyric acid** | **It can be used as an edible spice** | **C79312** | **C_4_H_8_O_2_** | **756.2** | **39.23** | **1.237** |
| **11** | **1-Pentanol** |  | **C71410** | **C_5_H_12_O** | **758.7** | **39.946** | **1.2571** |
| **12** | **2-butanone** | Mainly used to prepare cheese, coffee and banana flavor | **C78933** | **C_4_H_8_O** | **620.6** | **16.571** | **1.2428** |
| **13** | **3-Butenenitrile** | There is an unpleasant smell | **C109751** | **C_4_H_5_N** | **664** | **20.691** | **1.2657** |
| **14** | **1,2-Dimethoxyethane** | Has a strong ether-like smell | **C110714** | **C_4_H_10_O_2_** | **654.4** | **19.616** | **1.3232** |
| **15** | **1-butanol** | Has a smell similar to fusel oil | **C71363** | **C_4_H_10_O** | **678** | **22.482** | **1.3835** |
| **16** | **2-methylbutanal** |  | **C96173** | **C_5_H_10_O** | **674.6** | **22.035** | **1.3993** |
| **17** | **ethyl butanoate** | **Qingling intense sweet fruit flavor, high concentration fat odor** | **C105544** | **C_6_H_12_O_2_** | **789.8** | **49.35** | **1.5559** |
| **18** | ***** |  | **C109977** | **C_4_H_5_N** | **762.1** | **40.931** | **1.4352** |
| **19** | ***** |  | **C98011** | **C_5_H_4_O_2_** | **794.1** | **50.693** | **1.339** |
| **20** | **2,3-Butanediol** | It can be used as an edible spice | **C513859** | **C_4_H_10_O_2_** | **794.6** | **50.514** | **1.3683** |
| **21** | **alpha-Pinene** | Scent of pine, needle and resin | **C80568** | **C_10_H_16_** | **927.8** | **131.411** | **1.2083** |
| **22** | **2-heptanone** | A fragrant, stable liquid used as a spice material | **C110430** | **C_7_H_14_O** | **890.3** | **97.901** | **1.2604** |
| **23** | **isoamyl acetate** | Banana smell | **C123922** | **C_7_H_14_O_2_** | **874.7** | **87.039** | **1.302** |
| **24** | **hexanal** | Edible spices | **C66251** | **C_6_H_12_O** | **798.2** | **52.06** | **1.276** |
| **25** | **Heptanal** | Fruity flavor, used as an important ingredient in synthetic spices | **C111717** | **C_7_H_14_O** | **893** | **99.934** | **1.3583** |
| **26** | **Allylacetic acid** | Essence and fragrance | **C591800** | **C_5_H_8_O_2_** | **898.8** | **104.689** | **1.4389** |
| **27** | **hexanenitrile** | It has a very unpleasant smell | **C628739** | **C_6_H_11_N** | **874.8** | **87.138** | **1.5966** |
| **28** | **isoamyl acetate** |  | **C123922** | **C_7_H_14_O_2_** | **875** | **87.291** | **1.7424** |
| **29** | **2-acetylfuran** | It has sweet, almond, nut, roasted and smoky aromas | **C1192627** | **C_6_H_6_O_2_** | **921.9** | **125.596** | **1.4484** |
| **30** | **5-Methylfurfural** | It has a sweet caramel taste | **C620020** | **C_6_H_6_O_2_** | **958** | **163.983** | **1.3937** |
| **31** | **2-Heptenal, (E)-** |  | **C18829555** | **C_7_H_12_O** | **954.7** | **160.239** | **1.2525** |
| **32** | **alpha-Pinene** |  | **C80568** | **C_10_H_16_** | **926.9** | **130.48** | **1.6612** |
| **33** | **3-Furanmethanol** |  | **C4412913** | **C_5_H_6_O_2_** | **969.6** | **177.331** | **1.3481** |
| **34** | **hexanoic acid** | Has a smell similar to dry cheese | **C142621** | **C_6_H_12_O_2_** | **974.1** | **182.506** | **1.6272** |
| **35** | **1-Octen-3-ol** | Aroma of mushroom, lavender, rose and hay | **C3391864** | **C_8_H_16_O** | **975.3** | **183.853** | **1.7111** |
| **36** | **1-Heptanol** | perfumed | **C111706** | **C_7_H_16_O** | **972.8** | **180.994** | **1.7612** |
| **37** | **3-Octanone** |  | **C106683** | **C_8_H_16_O** | **992.8** | **204.111** | **1.7199** |
| **38** | **3-Octanol** | Colorless with a peculiar odor | **C589980** | **C_8_H_18_O** | **994.3** | **205.826** | **1.3918** |
| **39** | **2-Pentylfuran** | With bean fragrance, fruit fragrance, soil, green fragrance, vegetable fragrance | **C3777693** | **C_9_H_14_O** | **994.3** | **205.921** | **1.2445** |
| **40** | **alpha-Phellandrene** | Edible spices | **C99832** | **C_10_H_16_** | **975.5** | **184.126** | **1.2095** |
| **41** | **Pyrazine,2-ethyl-6-methyl-** | Nutty, roasted and sweet aromas. | **C13925036** | **C_7_H_10_N_2_** | **1011** | **224.914** | **1.2116** |
| **42** | **2(E)-hexenoic acid** |  | **C13419697** | **C_6_H_10_O_2_** | **1043.7** | **260.857** | **1.3057** |
| **43** | **3-Octanone** | Lavender fragrance | **C106683** | **C_8_H_16_O** | **991.2** | **202.296** | **1.3004** |
| **44** | **Strawberry furanone** | Aromas of baked caramel, fruity, burnt, caramel and pineapple | **C3658773** | **C_6_H_8_O_3_** | **1062** | **280.514** | **1.2123** |
| **45** | **Linalool** | With thick green with sweet wood green smell, floral, woody, fruity smell. | **C78706** | **C_10_H_18_O** | **1103.2** | **324.107** | **1.7506** |
| **46** | **2-phenylethanol** | Aroma of fresh bread, sweet rose - like flowers | **C60128** | **C_8_H_10_O** | **1121.9** | **343.891** | **1.3036** |
| **47** | **Diethyl butanedioate** | Have a special smell | **C123251** | **C_8_H_14_O_4_** | **1204.9** | **431.559** | **1.309** |
| **48** | **Methyl salicylate** | Strong aroma of Holly oil | **C119368** | **C_8_H_8_O_3_** | **1188.4** | **414.138** | **1.2117** |
| **49** | ***** |  | **C18999285** | **C_7_H_12_O_2_** | **1184** | **409.464** | **1.5891** |
| **50** | ***** |  | **C80717** | **C_6_H_8_O_2_** | **1030.3** | **246.307** | **1.4612** |
| **51** | **(E,E)-2,4-heptadienal** |  | **C4313035** | **C_7_H_10_O** | **1029.5** | **245.457** | **1.6317** |
| **52** | **1,8-Cineole** | Camphorlike aroma and cool taste | **C470826** | **C_10_H_18_O** | **1029.5** | **245.457** | **1.7219** |
| **53** | **Limonene** | It has a scent similar to lemon | **C138863** | **C_10_H_16_** | **1062.6** | **281.148** | **1.6718** |
| **54** | **Z-3-octen-1-ol** | It has a musty taste like mushrooms | **C20125842** | **C_8_H_16_O** | **1068.3** | **286.932** | **1.7283** |
| **55** | **Hexyl propanoate** | fruity | **C2445763** | **C_9_H_18_O_2_** | **1105.3** | **326.34** | **1.4377** |
| **56** | **Benzothiazole** | Smell like quinoline | **C95169** | **C_7_H_5_NS** | **1246.6** | **475.6** | **1.1607** |
| **57** | **Carveol** |  | **C99489** | **C_10_H_16_O** | **1246.1** | **475.099** | **1.6847** |
| **58** | **nerol** | **Fragrant with rose** | **C106252** | **C_10_H_18_O** | **1246.1** | **475.099** | **1.7155** |
| **59** | ***** |  | **C101973** | **C_10_H_12_O_2_** | **1242.1** | **470.838** | **1.9854** |
| **60** | **Geranyl propionate** | It smells of sweet grapes and roses | **C105908** | **C_13_H_22_O_2_** | **1478.4** | **720.375** | **1.2143** |
| **61** | **ortho-Guaiacol** | It has a special aromatic smell. | **C90051** | **C_7_H_8_O_2_** | **1093.5** | **313.527** | **1.2615** |
| **62** | **Ocimene** | Grassy, floral notes with neroli oil. | **C13877913** | **C_10_H_16_** | **1046** | **263.322** | **1.242** |
| **63** | **phenylacetaldehyde** | There is a strong scent of hosta flower | **C122781** | **C_8_H_8_O** | **1040.4** | **257.355** | **1.2569** |
| **64** | **2,3,5-trimethylpyrazine** | Used for cocoa, coffee and other baked food flavor | **C14667551** | **C_7_H_10_N_2_** | **1015.1** | **229.455** | **1.1644** |
| **65** | **Ethyl isovalerate** |  | **C108645** | **C_7_H_14_O_2_** | **842.7** | **69.451** | **1.2714** |
| **66** | **Dimethyl disulfide** | It has a foul smell | **C624920** | **C_2_H_6_S_2_** | **740.3** | **34.957** | **1.1574** |
| **67** | **5-Octanolide** | Creamy, coconut and vanilla aromas | **C698760** | **C_8_H_14_O_2_** | **1296.9** | **528.663** | **1.2907** |
| **68** | **geranyl formate** |  | **C105862** | **C_11_H_18_O_2_** | **1294.6** | **526.242** | **1.8481** |
| **69** | **Pentanoic acid** | There is an unpleasant smell | **C109524** | **C_5_H_10_O_2_** | **881.8** | **91.714** | **1.2297** |
| **70** | **Geraniol** | With mild, sweet rose smell, bitter taste | **C106241** | **C_10_H_18_O** | **1245.6** | **474.573** | **1.2143** |
| **71** | **2-methoxy-4-vinylphenol** | **It has a strong aroma of spices, cloves and fermentation, with a flavour of fried peanuts** | **C7786610** | **C_9_H_10_O_2_** | **1310.2** | **542.734** | **1.2173** |
| **72** | **2,6-Dimethoxyphenol** |  | **C91101** | **C_8_H_10_O_3_** | **1347.8** | **582.402** | **1.2098** |
| **73** | **Styrene** | perfumed | **C100425** | **C_8_H_8_** | **876.1** | **87.974** | **1.4138** |
| **74** | **2-heptanone** | It has a fruity aroma similar to pear | **C110430** | **C_7_H_14_O** | **888.6** | **96.679** | **1.6266** |
| **75** | **ethyl pentanoate** | It has an apple-like fruity aroma | **C539822** | **C_7_H_14_O_2_** | **880.9** | **91.101** | **1.6754** |
| **76** | **3-Methylpentanoic acid** | Sour herbal scent with hints of green | **C105431** | **C_6_H_12_O_2_** | **954.5** | **160.068** | **1.2831** |
| **77** | **2-acetylpyrazine** | Burst corn flower flavor, baked flavor | **C22047252** | **C_6_H_6_N_2_O** | **1030.7** | **246.741** | **1.546** |
| **78** | Acetophenone | It has a pleasant aroma | C98862 | C_8_H_8_O | 1043.3 | 260.61 | 1.594 |
